# New insight into the role of fibroblasts in the epithelial immune microenvironment in the single-cell era

**DOI:** 10.3389/fimmu.2023.1259515

**Published:** 2023-09-22

**Authors:** Liangzhe Wang, Bo Wang, Erwen Kou, Lin Du, Yuanjie Zhu

**Affiliations:** Department of Dermatology, Naval Medical Center, Naval Medical University, Shanghai, China

**Keywords:** fibroblasts, epithelial immune microenvironment, inflammatory skin diseases, single-cell RNA sequencing, spatial transcriptomics, cell crosstalk, prurigo nodularis

## Abstract

The skin is exposed to environmental challenges and contains heterogeneous cell populations such as epithelial cells, stromal cells, and skin-resident immune cells. As the most abundant type of stromal cells, fibroblasts have been historically considered silent observers in the immune responses of the cutaneous epithelial immune microenvironment (EIME), with little research conducted on their heterogeneity and immune-related functions. Single-cell RNA sequencing (scRNA-seq) and spatial transcriptomics (ST) have overcome the limitations of bulk RNA sequencing and help recognize the functional and spatial heterogeneity of fibroblasts, as well as their crosstalk with other types of cells in the cutaneous EIME. Recently, emerging single-cell sequencing data have demonstrated that fibroblasts notably participate in the immune responses of the EIME and impact the initiation and progression of inflammatory skin diseases. Here, we summarize the latest advances in the role of fibroblasts in the cutaneous EIME of inflammatory skin diseases and discuss the distinct functions and molecular mechanisms of activated fibroblasts in fibrotic skin diseases and non-fibrotic inflammatory skin diseases. This review help unveil the multiple roles of fibroblasts in the cutaneous EIME and offer new promising therapeutic strategies for the management of inflammatory skin diseases by targeting fibroblasts or the fibroblast-centered EIME.

## 1 Introduction

The skin contains heterogeneous cell populations, such as epithelial cells, skin-resident immune cells, and stromal cells, and acts as both a physical barrier and an immune organ that can defend against external damage and adverse factors (1, 2). Based on the current knowledge of skin structure, immune responses predominantly develop in the epithelial immune microenvironment (EIME), situated in the epidermis and papillary dermis of the skin (3, 4). Facing ever-changing internal and external stimuli, the cutaneous EIME develops an intricate system for immune responses, including the proliferation and differentiation of multiple cell types and the local interactions among activated immune cells, keratinocytes, and stromal cells in the EIME (4). Disruptions in the cutaneous EIME lead to dysregulated immune responses, inducing various inflammatory skin disorders.

Fibroblasts, characterized by collagen alpha-1 chain and decorin, are the major type of stromal cells in the cutaneous EIME and can be spatially categorized into papillary and reticular fibroblasts (5–7). Fibroblasts are the fundamental cellular component supporting the cutaneous framework because they can produce collagens to form the extracellular matrix (ECM) (8, 9). Historically, fibroblasts have been regarded as silent observers occupying a secondary role within the cutaneous EIME. Studies on the heterogeneity and immune-related functions of fibroblasts are limited (10, 11). Recently, the newly developed single-cell technologies have significantly facilitated our exploration of the morphological and functional heterogeneity of dermal fibroblasts, including single-cell RNA sequencing (scRNA-seq) and spatial transcriptomics (ST), which can recognize unique alterations in gene expression for each cell and provide quantitative visualization of the distribution of gene expression within tissue sections (12–15). Emerging evidence has revealed the dynamic changes and specific functions of fibroblasts in the cutaneous EIME during inflammation progression (16–18).

**Figure 1:**
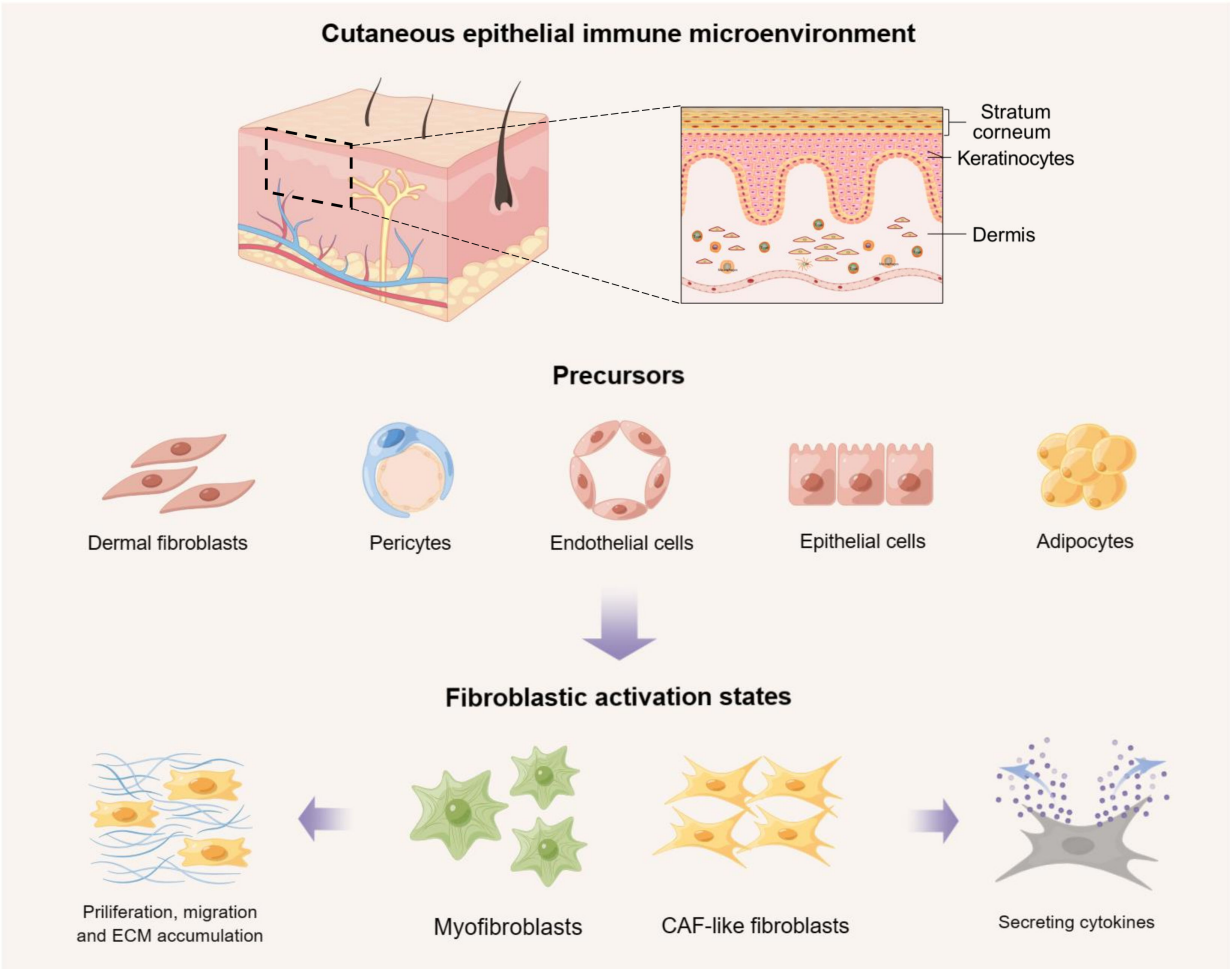
The activation states of fibroblasts and their precursors in the cutaneous epithelial immune microenvironment (EIME). Under different pathological conditions, such as wound healing, malignancy, or other inflammatory disorders, normal fibroblasts and other types of cells in the cutaneous EIME, including epithelial cells, endothelial cells, pericytes, and adipocytes, can be activated to develop into myofibroblasts or cancer-associated fibroblasts (CAFs). These activated fibroblasts can result in the abnormal accumulation of extracellular matrix (ECM), or secrete cytokines to interact with other types of cells. Created with Figdraw.

Under different pathological conditions, such as wound healing, malignancy, or other inflammatory disorders, normal fibroblasts can be activated and secrete cytokines to interact with other cells. Fibroblasts and other types of cells in the cutaneous EIME, such as epithelial cells, endothelial cells, pericytes, and adipocytes, can develop into myofibroblasts, or cancer-associated fibroblasts (CAFs) which are a heterogenous population of activated fibroblasts playing key roles in tumor microenvironment and affecting tumor proliferation, metastasis, and chemotherapy resistance (**Figure 1**) (19, 20). The activation states of fibroblasts are closely involved in the pathogenesis of diverse inflammatory skin diseases. Based on the distinct roles of fibroblasts in the cutaneous EIME, we divided inflammatory skin diseases into two major categories: (I) fibrotic skin diseases like keloid and systemic sclerosis (SSc), in which fibroblasts act as hallmark cells (21–23); and (II) non-fibrotic inflammatory skin diseases like psoriasis, atopic dermatitis (AD), vitiligo, and systemic lupus erythematosus (SLE), in which fibroblasts act as fundamental and active participants in the immune response by interacting with other types of cells in the cutaneous EIME (24–27).

In this review, we summarize the latest advances in the role of fibroblasts in the cutaneous EIME of inflammatory skin disorders and discuss the distinct functions and potential molecular mechanisms of activated fibroblasts in fibrotic skin diseases and non-fibrotic inflammatory skin diseases. We aim to unveil the multiple roles of fibroblasts in the cutaneous EIME and explore new promising therapeutic strategies targeting fibroblasts or fibroblast-centered EIME in inflammatory skin diseases.

## 2 Fibroblasts act as hallmark cells in fibrotic skin diseases

Fibrotic skin diseases, including local and systematic fibrotic diseases, are characterized by the excessive accumulation of fibroblasts and the abnormal buildup of the ECM and exert a major healthcare burden worldwide (28, 29). Fibroblasts act as the hallmark cells in fibrotic skin diseases. Under pathological conditions, normal fibroblasts and other types of cells can be stimulated to develop into fibroblastic activation states, resulting in excessive collagen accumulation and abnormal tissue fibrosis (19, 30). This section summarizes recent studies on the functions and underlying molecular mechanisms of fibroblasts in various fibrotic skin diseases to provide potential targets for treating refractory fibrotic skin diseases.

### 2.1 Local fibrotic skin diseases

Keloid is a typical local fibrotic skin disorder characterized by hypervascularity and excessive accumulation of the ECM (31, 32). Associated with progressive tissue fibrosis and a high recurrence rate, keloid leads to heavy burden and psychological issues for patients. Abnormal activation and excessive proliferation of fibroblasts are the core pathological manifestations in keloid. However, the underlying mechanisms and molecular changes remain unclear.

In a recent study, Yang et al. conducted a scRNA-seq analysis on dermis tissues from keloid and normal scar samples. They obtained transcriptomes of 40,655 cells (keloid: 21,488; normal scar: 19,167) and identified 13 fibroblast subclusters with further heterogeneity (17). Among the identified fibroblast subsets, a notable increase in the proportion of mesenchymal fibroblasts was observed in keloids compared to regular scars, along with a consistent upregulation of genes related to ECM formation, such as collagen type I alpha 1/2 (COL1A1/2), periostin (POSTN) and fibronectin 1 (FN1). Although prior research has documented the increased expression of certain proteins, including POSTN and FN1, the precise cellular origins of these proteins were uncertain. The use of scRNA-seq offers a valuable approach to unveil the cell-specific molecular alterations and mechanisms involved in keloid pathogenesis, a task that conventional bulk RNA sequencing and microarray techniques have struggled to achieve. In that study, keloid exhibited marked increases in the interaction between transforming growth factor-β (TGFβ) and its receptor as well as the interaction between POSTN and integrin alpha-V (ITGAV)/ITGB5 compared to normal scars, suggesting that the TGFβ pathway and POSTN may actively participate in the initiation and progression of keloid. These results were further validated through immunofluorescence staining assay. Consequently, inhibiting mesenchymal fibroblasts by blocking the TGFβ pathway or targeting POSTN might potentially serve as viable therapeutic approaches for treating keloid. The notable upregulation of mesenchymal fibroblasts and ECM-related genes, namely COL1A1, POSTN, COL1A2, FN1 and ASPN, in keloid was similarly documented in a distinct scRNA-seq study conducted by Huang et al. (33). Additionally, the researchers observed that mesenchymal fibroblasts can promote the differentiation and proliferation of macrophages, significantly contributing to the macrophage-centered regulatory network within the EIME of keloid. These findings substantially advanced our comprehension of the immunological characteristics of keloid.

**Figure 2:**
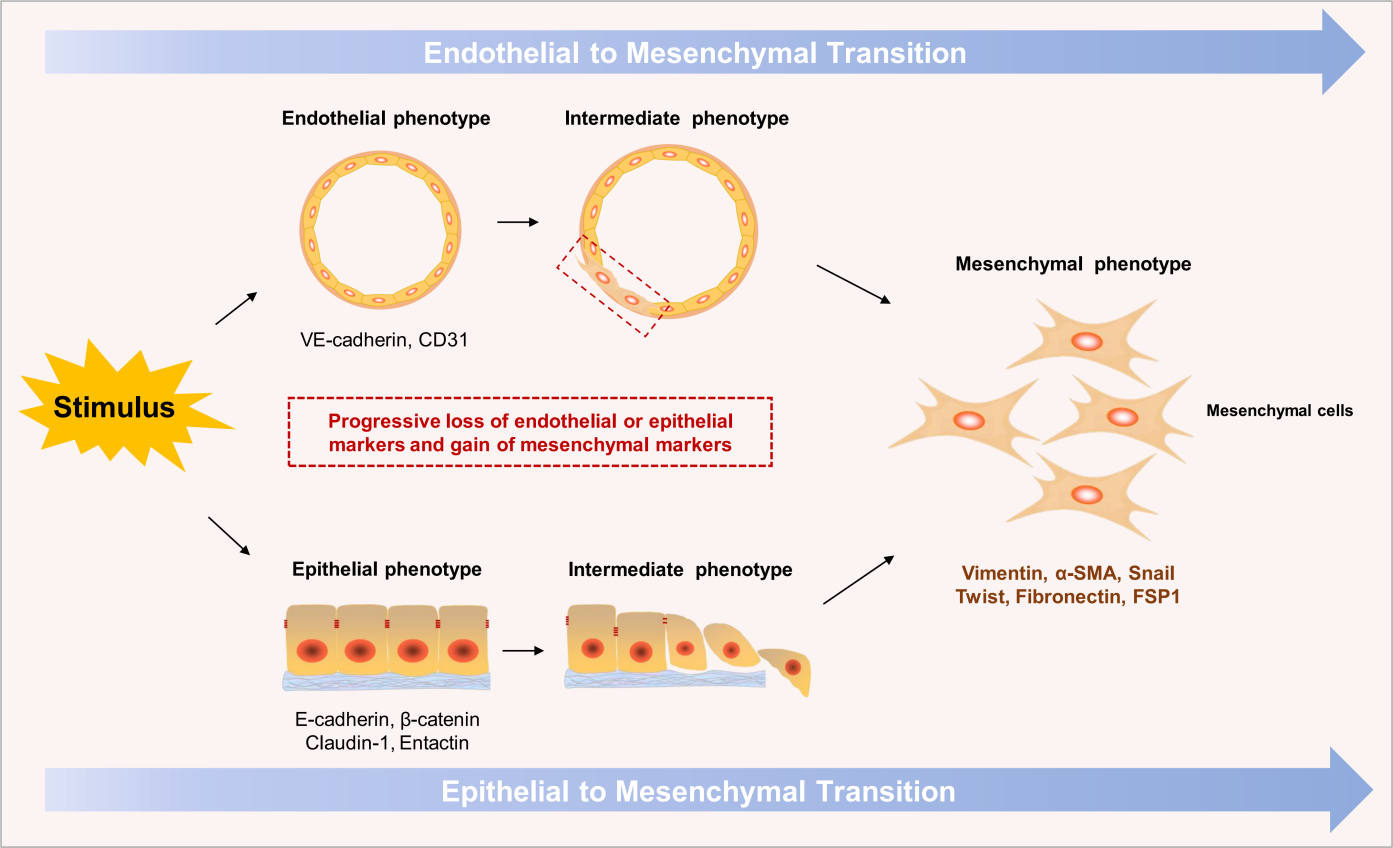
Sketch map of the endothelial to mesenchymal transition (EndoMT) and epithelial to mesenchymal transition (EMT) processes. In response to diverse inflammatory stimuli, endothelial cells or epithelial cells can be activated, transdifferentiating into mesenchymal cells, known as EndoMT or EMT respectively. These intrinsic processes entail the gradual loss of endothelial/epithelial markers, including CD31, vascular endothelial (VE)-cadherin or epithelial (E)-cadherin, and β-catenin, and the acquisition of mesenchymal markers, including fibroblast-specific protein (FSP1) and alpha-smooth muscle actin (α-SMA).

As the key contributor to keloid, mesenchymal fibroblasts can originate from several types of cells, such as normal fibroblasts, adipocytes and endothelial cells. Endothelial-to-mesenchymal transition (EndoMT) is an intrinsic transdifferentiated process that remarkably participates in the occurrence and development of tissue fibrosis under different pathological conditions (34, 35). The EndoMT process involves the reduction of specific endothelial markers, such as CD31 and vascular endothelial (VE)-cadherin, and the acquisition of mesenchymal markers, such as fibroblast-specific protein (FSP1) and alpha-smooth muscle actin (α-SMA) (**Figure 2**). Endothelial cells (ECs) can be stimulated by TGFβ, secreted by activated immune cells, and transdifferentiate into myofibroblasts or other overactive fibroblasts, leading to collagen accumulation and tissue fibrosis. For a long time, EndoMT has been commonly observed in cardiac fibrosis and kidney fibrosis, yet few studies have been conducted on its occurrence in skin fibrosis. Lee et al. recently performed a scRNA-seq analysis on 35,424 cells from two keloid samples and five normal skin samples. They use the single-cell data from a previously published cohort as normal skin control (36). Remarkably, mesenchymal activation, marked by dysregulated TGFβ/Smad signaling, was widely observed in keloid ECs, which is a characteristic of EndoMT. Moreover, the integration of ST allowed them to observe that the disease-related mesenchymal fibroblasts predominantly clustered around the endothelial regions and colocalized with the keloid ECs. These findings further suggested the potential involvement of fibroblast-EC communication and the EndoMT process in the pathogenesis of keloid. However, given the relatively limited sample size in this study and the lack of extensive prior research on the EndoMT process in keloid, further investigation is necessary to fully understand the impact of EndoMT on the occurrence and progression of keloid.

Endothelin-1 (ET-1), a powerful vasoactive peptide responsible for regulating vascular tone and produced by ECs, was observed to be highly expressed in keloid (37). Notably, the RhoA/Rho-kinase (ROCK) pathway can be activated by ET-1 to promote myofibroblast differentiation and ECM accumulation in the dermis, thereby markedly participating in the pathophysiology of abnormal scar/keloid formation. Therefore, targeting the expression of ET-1 or blocking the RhoA/ROCK pathway may provide powerful approaches for inhibiting ET-1-induced keloid (38).

CAF-like fibroblasts, with the increased expression of CAF-related genes, such as COL11A1, POSTN, WNT5A, and ASPN, is another key subset enriched in fibrotic diseases (33, 39). This fibroblast subset can originate from epithelial cells though the epithelial-to-mesenchymal transition (EMT) process (**Figure 2**) or the activation of normal fibroblasts under various inflammatory stimuli (30, 40, 41). Several recent studies have confirmed that CAF-like fibroblasts can remodel and boost the accumulation of the ECM or interact with other types of cells, resulting in the progression and expansion of fibrotic lesions in the EIME (42–44). These findings help us understand the mechanisms of tissue fibrosis and provide potential new targets for preventing and treating keloid and other skin diseases characterized by fibrosis.

### 2.2 Systematic fibrotic diseases involving the skin

Systemic sclerosis is a complicated disorder characterized by excessive fibrosis of the skin and multisystem organs. Continuous activation of fibroblasts into myofibroblasts is the core process in the pathogenesis of SSc (45, 46). However, the specific origins of myofibroblasts and their molecular changes in the skin of SSc patients remain uncertain. Recently, Lafyatis et al. performed a scRNA-seq analysis on mid-forearm skin biopsies from 12 patients with SSc and 10 healthy controls (47). They identified a new fibroblast subcluster mainly in SSc skin, named SFRP2^hi^ (expressing high levels of SFRP2) PRSS23^+^ fibroblasts, which might be associated with the ECM and extracellular structure organization. Moreover, the researchers used pseudotime analysis to ascertain the differentiation trajectory of cells and demonstrated a linear progression from SFRP2^hi^ WIF^+^ to SFRP2^hi^ PRSS23^+^ WIF1^−^ and to SFRP2^hi^ PRSS23^+^ SFRP4^+^ fibroblasts during the progression of SSc. Additionally, a series of transcription factors (TFs) implicated in myofibroblast differentiation was elucidated, including fos-like antigen 2 (FOSL2), runt-related transcription factor 1 (RUNX1), signal transducer and activator of transcription (STAT1), forkhead box prote1 (FOXP1), and interferon regulatory factor 7 (IRF7). These findings not only revealed a significant shift in fibroblast phenotypes but also unveiled the underlying alterations in gene expression in SSc skin, which can broaden our comprehension of the pathogenesis of SSc and help explore the origin and differentiation of myofibroblast in other fibrotic diseases.

The involvement of EndoMT in dermal fibrosis of SSc was determined through *in vitro* and *in vivo* experiments (48). CD31/α-SMA and VE-cadherin/α-SMA colocalization was markedly higher in SSc lesional dermis than in healthy skin dermis. Additionally, the ECs in SSc lesions showed a functional phenotype similar to that of myofibroblasts, indicating the potential participation of EndoMT in the progression of SSc. Furthermore, *in vitro* experimental results indicated that healthy dermal microvascular ECs (H-dMVECs) exposed to SSc sera developed a myofibroblast-like appearance, marked by a decrease in endothelial indicators and the activation of mesenchymal indicators. This phenotypic transition of H-dMVECs treated with SSc sera was consistent with that of H-dMVECs treated with TGFβ, a universally acknowledged stimulus for EndoMT, further suggesting the key role played by EndoMT in the progression of SSc. These studies revealed the close link between EC dysfunction and the progression of fibrosis, and thus, inhibiting EndoMT may be an effective approach for managing skin fibrosis in patients with SSc.

### 2.3 Other inflammatory skin diseases with focal fibrosis

In addition to conventional fibrotic skin disorders, certain inflammatory skin conditions exhibit focal fibrosis, such as prurigo nodularis (PN). As a chronic inflammatory skin disorder, PN is characterized by stubborn pruritic firm nodules on the extremities and trunk and has recently been associated specifically with the follicular epithelium in the cutaneous EIME (49–51). In the epidermis, it presents as orthohyperkeratosis, hypergranulosis, and acanthosis, occasionally reaching the extent of pseudoepitheliomatous hyperplasia (52). However, recent studies have provided new evidence indicating the unique fibroproliferative characteristics of PN compared with other inflammatory skin diseases, such as psoriasis and atopic dermatitis (23, 53). That reminds us the activated fibroblasts might potentially play a key role in the formation of PN lesions, which is consistent with its clinical features of firm fibrotic nodules.

Gudjonsson et al. conducted a scRNA-seq analysis on 72,782 cells from the skin of 15 healthy donors and the nonlesional and lesional skin of 6 patients with PN (54). They observed the presence of a specific fibroblast subset in the papillary dermis of PN skin, characterized by elevated expression levels of COL11A1, POSTN, and serine protease 23 (PRSS23). This subset, identified as COL11A1^+^ fibroblasts, demonstrated distinct profibrotic properties in the cutaneous EIME of PN. Additionally, the researchers also found that the receptor of interleukin-31 (IL-31), which is a favorable immune mediator in PN, was expressed in diseased fibroblasts, and therapy targeting IL-31R (Nemolizumab) could revert the changes in PN lesions. This finding provides convincing evidence of the critical role of immune-fibroblast crosstalk in the pathogenesis of PN. Remarkably, that study also used ST to ascertain the spatial heterogeneity and cell-cell interaction of fibroblasts in the cutaneous EIME. The incorporation of suitable controls and the integration of ST and scRNA-seq enhance the reliability and importance of these findings.

A separate single-cell analysis conducted by Kwatra et al. revealed a significant increase in CAF-like (WNT5A^+^ POSTN^+^) fibroblasts within PN lesions compared with nonlesional skin of PN patients (55). Moreover, ligand-receptor analyses demonstrated that the WNT5A and POSTN expressed by fibroblasts could frequently interact with the neuronal receptors MCAM and ITGAV in PN lesions, further suggesting the potential role of the fibroblast-neuronal axis in the initiation and progression of PN. However, this study solely focused on patients diagnosed with PN, without incorporating healthy individuals as a control group. Therefore, further extensive research is necessary to acquire a more comprehensive and persuasive understanding of the specific roles of fibroblasts in the development of PN.

The above studies at single-cell level enhanced our understanding of the roles of morphologically and functionally heterogeneous fibroblasts in the pathogenesis of PN. For a long time, most studies on prurigo nodularis focused on “itch”, and limited studies were conducted on firm “nodules”. Hence, exploring fibroblast-related molecular mechanisms of firm nodules may provide optional and promising strategies for treating refractory PN.

## 3 Activated fibroblasts-entangled crosstalk in non-fibrotic inflammatory skin diseases

In addition to playing a central role in fibrotic skin diseases, fibroblasts also actively participate in the cutaneous EIME of non-fibrotic inflammatory skin diseases, such as psoriasis, AD, SLE, and vitiligo, mostly by frequent crosstalk with immune cells, epithelial cells, and others. Single-cell data provides a unique opportunity to analyze cell-cell communication mediated by ligand-receptor interactions. This section discusses the new insight into the role of crosstalk between fibroblasts and multiple types of cells in non-fibrotic inflammatory skin diseases to discover novel and powerful targets for managing diverse inflammatory skin diseases.

### 3.1 Fibroblast-immune cell crosstalk

Psoriasis is one of the most common immune-regulated skin diseases, exerting a substantial medical burden on patients and society (56, 57). Previous studies have confirmed that psoriasis is closely related to T-cell immune dysregulation, and IL-17 and IL-23 are regarded as the core initiators of the pathogenesis of psoriasis (58). Recently, researchers have combined scRNA-seq and ST to identify several novel fibroblast subsets in the cutaneous EIME of psoriasis that may drive and participate in the progression of psoriasis. Meanwhile, the crosstalk between fibroblasts and immune cells has also been demonstrated as the key contributor to psoriatic inflammation.

Hu et al. conducted a scRNA-seq analysis on full-thickness skins obtained from the psoriatic lesions of 3 patients and the corresponding regions of 3 healthy individuals, and a total of 24,234 cells were further clustered into 35 cellular subsets (59). They analyzed cell-specific gene expression and observed that the expression of MHC molecules, including HLA-A/B/C and HLA-DRA, was greatly increased on the surface of fibroblasts under psoriatic-inflammation conditions, which can stimulate the activation of T and NK cells by interactions with CD4, CD8, and CD94 (60, 61). Additionally, fibroblasts in psoriatic lesions showed a significant increase in the release of key chemokines and cytokines, including C-C motif chemokine ligand 26 (CCL26), IL-6, leukemia inhibitory factor (LIF), IL-17B, CCL19, and stromal cell-derived factor 1 (SDF-1), which greatly enhanced the accumulation of a variety of immune cells around fibroblasts. The release of a series of cytokines further confirmed the proinflammatory roles exerted by fibroblasts. Notably, these key molecules, such as CCL26, IL-6, IL-17B, and CCL19, have been previously considered active participants in the inflammatory process and are solely secreted by immune cells. In contrast, single-cell data help to reveal that fibroblasts can also secrete inflammatory cytokines and contribute to the immune responses of psoriasis.

Perivascular fibroblasts, an important subtype of fibroblasts, are widely distributed in human tissues and involved in various inflammatory diseases, such as myocardial infarction and fibrotic diseases. In contrast, their roles in psoriasis are rarely studied. By combining scRNA-seq and ST, a novel subset of PDGFRβ^+^ perivascular fibroblasts was identified in psoriatic lesions. This subset exhibited a remarkable increase in the release of IL-17B and activation of dendritic cells (DCs). The presence of PDGFRβ^+^ fibroblasts might be relevant to the surface expression of CD80/86 proteins in DCs, thereby promoting the progression of psoriatic inflammation (62). Nevertheless, the underlying mechanisms by which PDGFRβ^+^ fibroblasts secrete cytokines and affect the expression of CD80/86 proteins in DCs remain to be elucidated. These findings confirmed that fibroblasts could interact with immune cells by secreting a series of inflammatory factors and significantly regulate the immune responses in the EIME of psoriasis, providing promising targets and pathways for treating psoriasis.

Atopic dermatitis is a refractory inflammatory skin disease with clinical features of dry skin, intense itching, and eczema-like rashes (63–66). Although the etiology and underlying mechanisms have not yet been fully elucidated, AD has been consistently characterized by a robust stimulation of TH2 immune dysfunction in skin lesions and normal skin (67, 68). Compared to conventional bulk transcriptomics, single-cell analysis offer a more precise, comprehensive, and reliable characterization of immune subsets in AD. Researchers have identified novel fibroblast subpopulations in the cutaneous EIME that might contribute to the progression of inflammation and pathological disruption of AD.

Recently, Guttman et al. conducted a comprehensive scRNA-seq analysis on 39,042 cells obtained from lesional and nonlesional skin samples from 5 patients with moderate-to-severe AD and from healthy individuals. The inclusion criteria for the patients were strictly defined. Using cell lineage markers, the researchers identified a novel COL6A5/18A1^+^ fibroblast subset uniquely distributed in the AD lesions, associated with a higher release of the CCL2, CCL19, and IL-32 (25). COL6A5, also present in other locations of individuals with allergic conditions, has been identified as a gene involved in the development of AD (69). The potential contribution of COL6A5 in AD may be attributed to the creation of unstable heterotrimers, resulting in abnormal fibroblast adhesion and barrier dysfunction (70). COL18A1 can strongly bind to the ECM component and potentially disrupt and destabilize the ECM in AD lesions. Interestingly, they also observed that CD3^+^ T cells accumulated mostly around COL6A5/18A1^+^ fibroblasts, indicating that these fibroblasts may be involved in the recruitment and organization of T cells by secreting a series of cytokines. Additionally, as the best indicator of TH2 immune responses (71, 72), CCL26 upregulated the expression in COL6A5/18A1^+^ and MFAP5^+^ FBN1^+^ fibroblasts, further suggesting the critical roles of these fibroblast subsets in the TH2 immune responses of AD (25). As an important chemokine involved in several inflammatory processes, CCL26 has been widely studied and might be a favorable target for addressing inflammatory skin disorders (73, 74). Nevertheless, the cell-cell interactions and underlying pathways by which CCL26 contributes to the pathogenesis of AD remain unclear and need further investigation. In addition to COL6A5/18A1^+^ fibroblasts, AD lesions also exhibited higher infiltration of CCL2^+^ fibroblasts, with consistent patterns observed for CCL19 and POSTN. The CCL2^+^ fibroblasts are mostly distributed next to CD3^+^ T cells and can interact with macrophages and DCs via the receptors CCR1 and CCR2, further suggesting the critical roles of CCL2^+^ fibroblasts in the recruitment and organization of T cells, macrophages, and DCs. Despite the relatively small sample size, this study represents the initial comprehensive scRNA-seq analysis covering all cell types and expression conditions within the skin tissues of AD patients compared to healthy individuals. Therefore, the findings in this research are reliable, representative, and of substantial value.

Combining scRNA-seq with a series of *in vivo* experiments, Ko et al. first identified a unique paired related homeobox-1 (Prx1^+^) fibroblast subpopulation in AD lesions in a classical experimental mouse model, in which the IKKB-NF-kB pathway was disrupted under homeostatic conditions (75). These Prx1^+^ fibroblasts can overexpress CCL11 and lead to skin inflammation of AD, which is characterized by the infiltration of eosinophils and followed by TH2 immune responses. The effectiveness of monoclonal antibody therapy against CCL11 in reducing eosinophilia and TH2 inflammation confirmed the proinflammatory effect of CCL11 (75). Additionally, the researchers also examined human AD samples and confirmed that the perturbation of IKKB-NF-kB could upregulate CCL11 in human AD fibroblasts, further suggesting the critical roles of Prx1^+^ fibroblasts and CCL11 in the pathogenesis of AD. However, as a key chemokine and medium in the crosstalk between fibroblasts and immune cells, CCL11 has never been studied in inflammatory skin diseases other than AD (76). Hence, the underlying pathway by which CCL11 stimulates eosinophil infiltration and the TH2 immune response in the human dermis deserves further investigation (77).

In previous studies, the C-X-C motif receptor (CXCR4)/C-X-C motif ligand 12 (CXCL12) axis was reported to participate in psoriasis-like inflammation both by promoting keratinocyte proliferation and recruiting T cells (78). Likewise, the upregulation of CXCL12 was also identified in AD lesions. Sun et al. reported that thymic stromal lymphopoietin (TSLP) could induce fibroblasts to produce CXCL12 and stimulate the trafficking and migration of natural killer T cells (NKT) via CXCR4, resulting in inflammation progression in AD skin lesions (79). Thus, blocking CXCR4/CXCL12 could also be a feasible treatment option for individuals with AD.

SLE is a severe inflammatory disease characterized by diverse clinical and immunopathological manifestations (80, 81). Dysregulated activation of immune cells and aberrant secretion of autoantibodies and proinflammatory cytokines participate in the pathogenesis of SLE (82, 83). As a critical type of lupus erythematosus (LE), cutaneous lupus erythematosus (CLE) has been commonly studied, and the cellular heterogeneity and underlying mechanisms in cutaneous lesions have been revealed at single-cell resolution (84–86). Recently, Zhao et al. reported the scRNA-seq data of 23 skin biopsy samples from 8 discoid lupus erythematosus (DLE), 10 SLE patients, and 5 healthy controls (85). The researchers observed that CXCL1^+^ fibroblasts and HLA^+^ fibroblasts were the most abundant fibroblast subtypes in cutaneous lesions, and CXCL1, known for its ability to facilitate the infiltration of immune cells, was highly expressed in these activated fibroblasts. Additionally, the receptors of fibroblast-secreted CCL19, CXCL12, and tumor necrosis factor superfamily member 13b (TNFSF13B), which participate in the immune responses of the cutaneous EIME, exhibited higher expression in the immune cells of LE cutaneous lesions compared with healthy skin. These findings were also validated by immunofluorescence staining assay, indicating that the crosstalk between fibroblasts and immune cells contributes to the pathological processes of lupus cutaneous lesions.

In addition to releasing cytokines and chemokines, researchers found that activated fibroblasts might interact with immune cells by presenting antigens. HLA-DRB1 and HLA-DRA1, typically marked in antigen-presenting cells, were found to be highly expressed in HLA^+^ fibroblasts, suggesting the potential role of HLA^+^ fibroblasts as nonclassical antigen-presenting cells in immune responses (59). Nevertheless, the underlying pathways by which HLA^+^ fibroblasts present antigens and the specific types of involved immune cells remain to be elucidated.

### 3.2 Fibroblast-epithelial cell crosstalk

Keratocytes are the most abundant cell type in the epidermis, chiefly involved in the immune responses in the cutaneous EIME (87, 88). Consistent with immune cells, abundant crosstalk between fibroblasts and keratinocytes has also been confirmed in the pathogenesis of psoriasis. Using a skin equivalent model containing both psoriatic and healthy cells, researchers observed that psoriatic fibroblasts could stimulate the excessive proliferation of healthy keratinocytes, which is the main pathological manifestation of psoriasis (89). Moreover, the epidermis isolated from psoriatic skin continued to be hyperproliferative for at least 15 days, with no inhibition by normal fibroblasts, further demonstrating the key roles played by psoriatic fibroblasts in keratocyte hyperproliferation (90). However, revealing the underlying molecular mechanisms and signaling pathways by which fibroblasts interact with keratinocytes is critical for studies on psoriasis.

More recently, Miossec et al. observed that fibroblasts from individuals with psoriasis exhibit a significant increase in proliferation and enhance the growth of keratinocytes through the expression of SDF-1, also named CXCL12 (90, 91). SDF-1, a protein specific to fibroblasts, possesses the ability to trigger the ERK pathway and acts as a growth factor to induce the proliferation of epidermal keratinocytes. In-depth studies also confirmed that the overexpression of SDF-1 could significantly increase the quantity of keratinocyte layers and the thickness of the epidermis (92). SDF-1 has been widely studied as a stable chemokine that induces the migration and activation of various cell types, and SDF-1/CXCR4 targeted therapies have been successfully developed and applied in diverse diseases (93, 94). Thus, although SDF-1 is less studied in psoriasis than in tumors, and the underlying molecular mechanisms remain unclear, SDF-1 is still a promising target in the cutaneous EIME to inhibit keratinocyte proliferation and subsequently treat or prevent the occurrence of psoriasis.

Fibroblasts can alter the proliferation and differentiation of keratinocytes in a series of inflammatory diseases, but their role in AD has not been fully elucidated (90, 95). Recent studies confirmed the contribution of crosstalk between fibroblasts and keratinocytes to the pathogenesis of AD in the cutaneous EIME. Through atopic-like organotypic culture (OTC) skin models, Gitta et al. first revealed that atopic fibroblasts could impact the characteristics of the epidermis by promoting cell proliferation and hindering the layering of keratinocytes (18). Furthermore, atopic fibroblasts significantly downregulate the expression of LIF, a key regulator of the cytokine cascade (96), and release several cytokines, including IL-4, IL-3, and IL-31. These cytokines can diminish filaggrin expression in keratinocytes, leading to dysfunctions of the epidermal barrier in AD skin (97, 98). Notably, filaggrin is a major protein in maintaining the normal function of the epidermal barrier, and the lack or dysfunction of filaggrin can result in a range of skin disorders (99, 100). Hence, the phenomenon that filaggrin expression in keratinocytes can be downregulated by atopic fibroblasts and restored by healthy fibroblasts offers promising strategies for treating AD.

### 3.3 Fibroblast-other cell crosstalk

Melanocytes are an unremarkable cell type situated at the basal layer of the epidermis, and they can secrete melanin to maintain the appearance of the skin and protect it from ultraviolet damage (101, 102). Vitiligo is a unique inflammatory skin disease in which the immune system targets melanocytes, leading to the formation of depigmentation (103, 104). However, whether fibroblasts participate in the immune responses in vitiligo remains unclear.

**Figure 3:**
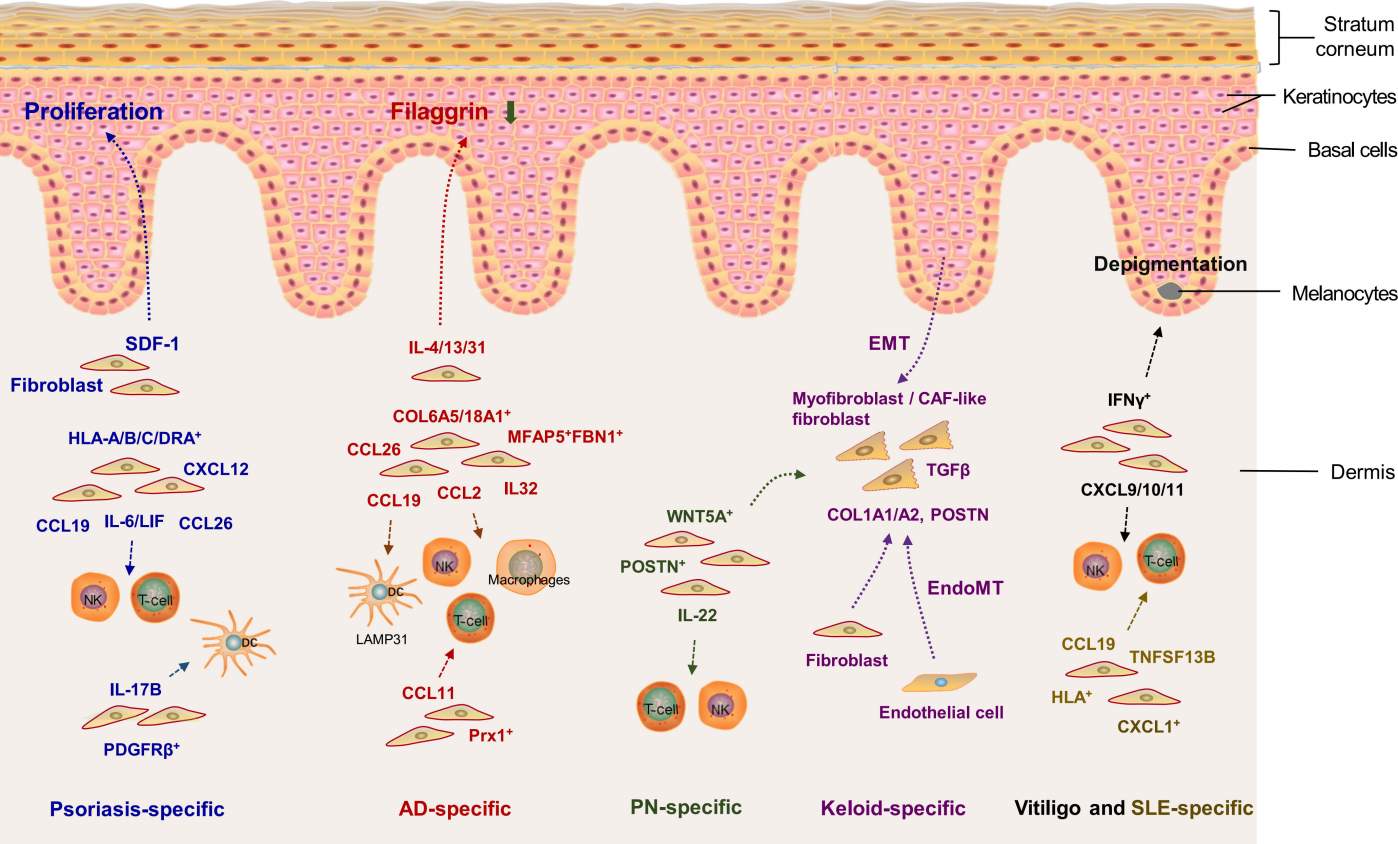
Crosstalk between fibroblasts and other types of cells in the cutaneous epithelial immune microenvironment (EIME) of inflammatory skin diseases. Blue, red, green, purple, black and golden words represent psoriasis-specific, atopic dermatitis (AD)-specific, prurigo nodularis (PN)-specific, keloid-specific, vitiligo-specific and systemic lupus erythematosus (SLE)-specific inflammation, respectively. SDF1, stromal-derived factor 1; TGF-β, transforming growth factor-β; CXCL, C-X-C motif ligand; CCL, C-C motif chemokine ligand; LIF, leukemia inhibitory factor; CAF, cancer-associated fibroblast; COL6A5, collagen type VI alpha 5; POSTN, periostin; TNFSF13B, tumor necrosis factor superfamily member 13b. IFNγ, interferon-γ; EMT, epithelial-mesenchymal transition; EndoMT, endothelial to mesenchymal transition.

Recently, Chen et al. conducted an extensive investigation into the distinct functions of fibroblast subsets in the pathogenesis of vitiligo (105). Using scRNA-seq, cell-type-specific knockouts, and engraftment experiments, the researchers demonstrated the importance of interferon γ (IFNγ) responsive fibroblasts in the recruitment and activation of CD8^+^ T cells. The CD8^+^ T cells subsequently attacked melanocytes, leading to the loss of melanocytes and skin depigmentation (105, 106). This subset of IFNγ-responsive fibroblasts and its pivotal role in the cutaneous EIME of vitiligo lesions have never been elucidated in prior research. Additionally, the researchers revealed that the activated IFNγ-responsive fibroblasts can recruit CXCR3^+^ T cells and interact with melanocytes by secreting chemokines CXCL9/CXCL10 in vitiligo lesions (105, 107). The CXCL9/CXCL10-CXCR3 axis is closely involved in the progression of vitiligo and deserves further attention. The highlight of this study lies in its comprehensive and logical experimental design. In addition to conducting scRNA-seq analysis on skin specimens obtained from vitiligo patients, the researchers employed a diverse range of *in vitro* and *in vivo* experiments, such as gene knockouts, engraftment experiments, and transwell migration assays, to investigate the specific functions of different fibroblast subsets in vitiligo. These comprehensive approaches enhance the credibility and validity of the research findings. Consequently, the triangulated crosstalk between fibroblasts, T cells, and melanocytes in the EIME of vitiligo provide promising methods and targeted strategies for preventing and treating vitiligo.

(Note: The identified fibroblast subsets and their crosstalk with other types of cells in the cutaneous EIME of diverse inflammatory skin disorders are illustrated in **Figure 3**)

## 4 Conclusions and future outlook

Thanks to the newly comprehensive analysis of the morphological and functional heterogeneity of fibroblasts by combining scRNA-seq and spatial transcriptomics, new insights can be obtained into the multiple roles of fibroblasts in the cutaneous EIME of inflammatory skin disorders. The present review discussed the distinct roles of fibroblasts in two major categories of inflammatory skin diseases.

In fibrotic skin diseases, the fibrous hyperproliferation and overaccumulation of the ECM caused by overactivated fibroblasts, as a hallmark, are crucial in the progression of these diseases. Therefore, strategies directly targeting fibroblasts or blocking the activation of fibroblasts are valuable for treating fibrotic skin diseases. In fact, new drugs or therapies targeting fibroblasts have emerged in recent years, especially in cancer research.

In the past few years, there has been a significant increase in the investigation of cancer-associated fibroblasts (108, 109). CAFs are commonly considered nonmutant cells within the tumor microenvironment that can significantly regulate cancer progression and metastasis (110, 111). Here, we summarize the current research on the roles of CAF-like fibroblasts in inflammatory skin disorders beyond the conventional understanding of CAFs in cancer research. We think CAFs are not a unique phenotype of tumors but a special phenotype or state of activated fibroblasts during wound healing or other inflammatory processes. Therefore, strategies targeting CAF-like fibroblasts may provide promising approaches for managing inflammatory skin diseases. Currently, although the phenotypes, origin, and functions of CAFs remain controversial, a series of novel drugs or biologics have been developed and prepared for anticancer clinical practice, such as galunisertib, JNJ-42756493, AMD3100, and PT630 (**Table 1**). These drugs can inhibit CAF activation or action by targeting a series of key molecules, such as fibroblast growth factor receptor (FGFR), TGFβ, CXCR4, ROCK, and fibroblast activation protein-α (FAP) (110, 123, 124). Given the crucial roles of CAFs/CAF-like fibroblasts in the cutaneous EIME of inflammatory skin diseases, these novel drugs targeting CAFs may also provide promising therapies for various inflammatory skin diseases with CAF-like fibroblasts, such as keloid, PN, and other diseases characterized by excessive fibrosis: this deserves further research.

**Table 1:** Current drugs that potentially target CAFs in clinical or preclinical trials (108, 110).

Drugs	Targets	Mechanisms	Status		Refs
Target CAF activation					
Galunisertib	TGFβ	Prevents CAF activation and immunosuppression	Phase III		(112)
Ruxolitinib	JAK-STAT3	JAK-STAT3 pathway inhibition	Phase II		(113)
JNJ-42756493	FGFR	Prevents CAF activation	FDA approved		(114)
IPI-926	Hedgehog	Hedgehog pathway inhibition	Preclinical		(115)
Target CAF action					
AT13148	ROCK	Reduces contractility	Phase II		(116)
AMD3100	CXCR4	Blocks the SDF1-CXCR4 interaction	Preclinical		(117)
Defactinib	FAK	Reduces signaling downstream of integrins	Clinical trials ongoing		(118)
Simtuzumab	LOXL2	Anti-crosslinking	Preclinical		(119)
FG-3019	CTGF	Blocks binding to receptors, including integrins	Preclinical		(120)
PEGPH20	Hyaluronic acid	ECM degradation to increase the access and efficacy of cytotoxic therapies and immunotherapies	Phase III completed		(121)
PT630	FAP	Blocks FAP^+^ CAF function, promoting T-cell function	Phase I		(122)

CAF, cancer-associated fibroblast; TGF-β, transforming growth factor-β; JAK, Janus kinase; STAT, signal transducer and activator of transcription; FGFR, fibroblast growth factor receptor; ROCK, RhoA/Rho-kinase; CXCR4, CXC-chemokine receptor 4; SDF1, stromal-derived factor 1; FAK, focal adhesion kinase; LOXL-2, lysyl oxidase-like 2; CTGF, connective tissue growth factor; FAP, fibroblast activation protein-α.

Activated fibroblasts can also release chemokines or other proinflammatory substances to interact with other types of cells in the cutaneous EIME (**Figure 3**). The crosstalk between fibroblasts and multiple types of cells significantly participates in the pathogenesis of fibrotic and non-fibrotic inflammatory diseases. It would be a valuable strategy to disrupt the crosstalk between fibroblasts and other cells for managing non-fibrotic inflammatory diseases. These findings at the single-cell level broaden our understanding of the roles of fibroblasts in the cutaneous EIME under inflammatory conditions and provide new targets and pathways to prevent and treat diverse inflammatory skin diseases (**Table 2**).

**Table 2:** Summary of potential targets of fibroblasts in the cutaneous EIME for the management of diverse inflammatory skin diseases.

Targets	Functions	Diseases	Refs
Fibrotic skin diseases (fibroblasts as hallmark cells)			
TGFβ/Smad signaling	Inducing mesenchymal fibroblasts and CAFs activation	Keloid, PN, SSc	(17, 36)
POSTN/ITGAV	Enhancing ECM formation and profibrotic effects	Keloid, PN	(17)
ET-1/ROCK pathway	Promoting myofibroblast differentiation and ECM accumulation	Keloid	(37)
PRSS23/IL-31	Key profibrotic molecules	PN	(54)
Non-fibrotic inflammatory skin diseases (fibroblasts interact with other cells)			
HLA-A/B/C and -DRA	Antigen presentation	Psoriasis, AD	(60)
CCL26	Classical type 2 chemokines	Psoriasis, AD	(25, 59)
CCL19	Recruiting DCs and T cells by CCR7	Psoriasis, AD, SLE	(25, 59, 85)
CCL2	Polarizing TH2 response by CCR2	AD	(25, 125)
IL-6/LIF	Key inflammatory cytokines	Psoriasis	(126)
IL-17B	Involved in surface expression of CD80 and CD86 protein in DCs	Psoriasis	(62)
CXCL12 (SDF-1)	Recruiting NKT and inducing TH2 responses via CXCR4/CXCL12 axes	Psoriasis, AD, SLE	(79, 92)
IL-4/IL-3/IL-31	Diminishing filaggrin expression in keratinocytes	AD	(97, 98)
CCL11	Inducing eosinophil infiltration and TH2 immune responses	AD	(92)
CXCL1/TNFSF13B	Inducing the infiltration and activation of immune cells	SLE	(85)
CXCL9/10/11	Recruiting CXCR3^+^ T cells to kill melanocytes	Vitiligo	(105)

TGF-β, transforming growth factor-β; CAF, cancer-associated fibroblast; POSTN, periostin; ITGAV, integrin alpha-V; ROCK, RhoA/Rho-kinase; ET-1, endothelin-1; PRSS23, serine protease 23; interleukin-31, IL-31; CCL, C-C motif chemokine ligand; LIF, leukemia inhibitory factor; CXCL, C-X-C motif ligand; CXCR4, CXC-chemokine receptor 4; SDF1, stromal-derived factor 1; IFNγ, interferon-γ; TNFSF13B, tumor necrosis factor superfamily member 13b. AD, atopic dermatitis; PN, prurigo nodularis; SLE, systemic lupus erythematosus.

Although single-cell technologies are available with perfect accuracy, we still need wide dynamic observation of the identified cell “subtypes”. The cell “subtypes” may indicate varying reactions to internal and external stimuli or damages instead of consistent functional specializations in a stable condition. This concept is similar to that established for a group of functionally unique subcategories of T cells, including Tregs, CD8^+^ T cells, CD4^+^ T cells, and double-negative T cells. Moreover, single-cell analyses via scRNA-seq and spatial transcriptomics cannot completely reflect the cellular protein levels or posttranslational protein modifications of molecular complexity and heterogeneity (127–129). Hence, the single-cell data should be analyzed more objectively and comprehensively, and other approaches should be integrated to reach a relatively precise conclusion.

## Author contributions

LW: Investigation, Resources, Writing – original draft. BW: Investigation, Resources, Writing – original draft. EK: Methodology, Writing – original draft. LD: Supervision, Visualization, Writing – review & editing. YZ: Conceptualization, Funding acquisition, Supervision, Writing – review & editing.
